# Hospital Meetings

**Published:** 1905-04-22

**Authors:** 


### HOSPITAL MEETINGS.

### THE EVELINA HOSPITAL.

The annual meeting of the Evelina Hospital took place on Wednesday, 12th inst., the Earl of Ancaster, president, in the chair.

The Chairman congratulated the Governors on the fact that, thanks to the kindness of an anonymous friend who sent a donation of £3,000, the debt to the bank was paid off, leaving a balance in hand of £441 17s. 9d.

The arrangement by which certain cases were sent to the Hospital for the Treatment of Hip Disease at Sevenoaks was an admirable one, since it not only benefited the children but relieved the hospital of four beds. Through the generosity of a friend the committee had been able to considerably enlarge the scope of the original and experimental scheme, and a far larger number would be dealt with during 1905 at various other country hospitals. This would be referred to in the next annual report, when the results of the scheme would be better known.

Mr. Wightman drew attention to the fact that the committee again urgently appealed for funds to enable them to enlarge the out-patient department. Each year the applicants for relief increased considerably, and the space was now quite inadequate. Plans of the necessary alterations and additions had been prepared, but until more money was forthcoming the committee did not feel justified in beginning the work. In a neighbourhood where poverty was extreme the legitimate demand upon this department was great. The hospital had sustained a severe loss in the retirement, on her marriage, of the matron, Miss Getz. Her place had been filled by Miss Goode, formerly assistant matron.

The report and accounts having been adopted, the usual votes of thanks closed the proceedings.

### THE MAGDALEN HOSPITAL.

The annual Court of Governors was held in the board-room of the hospital on the 12th instant, the Archbishop of Canterbury being in the chair.

Proceedings opened by the reading of the report by the Warden, the Rev. W. Watkins, which was unanimously adopted.

The Archbishop made an interesting and impressive speech, drawing attention to the fact that the work carried on in the hospital was intended to be recuperative and strengthening to the character, enabling the inmates to make a fresh start in the battle of life.

The Warden also made a short speech, and anxiously drew the attention of his audience to the heavy rates and taxes with which the charity was burdened. The hospital was now rated at £1,500, whereas at the last assessment it was only at £650.

The meeting closed with a vote of thanks to the Archbishop for presiding.

